# Synthesis of citric acid-modified resins and their adsorption properties towards metal ions

**DOI:** 10.1098/rsos.171667

**Published:** 2018-08-15

**Authors:** Xiong Liu, Longqi Xu, Yaqing Liu, Wenqi Zhou

**Affiliations:** 1 School of Chemistry and Chemical Engineering, Hunan University of Science and Technology, Xiangtan 411201, Hunan, People's Republic of China; 2 Hunan Provincial Key Laboratory of Controllable Preparation and Functional Application of Fine Polymers, Xiangtan 411201, People's Republic of China

**Keywords:** citric acid, resins, adsorption capacity, metal ions

## Abstract

Types of resins anchored on citric acid were synthesized and identified. The citric acid-modified resins PS-CA, PS-O-CA and PS-N-CA were synthesized by anchoring citric acid on PS-Cl, PS-OH and PS-NH_2_, respectively. The PS-CA, PS-O-CA and PS-N-CA were used to adsorb Fe^3+^, Al^3+^, Cu^2+^, Pb^2+^, Cd^2+^ and Hg^2+^. The influences of pH, adsorption time and metal ion concentration on the adsorption capacities of the resins were investigated. After optimization, PS-CA was a good adsorbent to Fe^3+^, Cu^2+^, Pb^2+^ and Cd^2+^ with *q_m_* values of 143.9 mg g^−1^, 77.4 mg g^−1^, 18.9 mg g^−1^ and 119.9 mg g^−1^, respectively. PS-N-CA was a good adsorbent to Al^3+^ and Hg^2+^ with *q_m_* values of 176.6 mg g^−1^ and 114.9 mg g^−1^, respectively. The adsorption kinetics and adsorption isotherm experiments indicated that the pseudo-first-order rate equation was more appropriate for characterizing the kinetic data and the Langmuir model was more suitable for fitting the equilibrium data. The reusability of the citric acid-modified resins was also evaluated and these resins exhibited considerable reusability.

## 1. Introduction

The presence of metal ions in water resources is one of the most common environmental pollutions in developing countries [1]. Metal ions are steadily accumulated in the living environment and cause serious damage to the nervous system, reproductive system, kidney, liver and brain of animals and human beings [2,3]. In order to remove metal ions, several methods, such as oxidation, precipitation, ion-exchange and adsorption, are widely adopted in water treatment [4–7]. Among them, the adsorption method is of great interest because of its easy operation and low cost [8]. Chelating resins acting as adsorbents have been widely studied recently for their good combining capacities and adsorption capacities towards metal ions [9]. Furthermore, metal ions adsorbed on chelating resins could also be efficiently desorbed, which makes resins recyclable. Functional groups, such as –OH, −COOH and −NH_2_, in resins are the main acting sites that could combine with metal ions. For instance, Mohammad *et al*. synthesized a new chelating resin functionalized with 2,3-dihydroxybenzoic acid and found that this new chelating resin exhibited good adsorption capacity towards Fe^3+^ [9]. Jermakowicz-Bartkowiak *et al*. have synthesized aminoguanidyl group-modified resins and these resins were good adsorbents for gold [10]. These works revealed that anchoring appropriate functional groups on resins is the key procedure to obtain chelating resins with high adsorption capacities towards metal ions.

**Figure 1.**
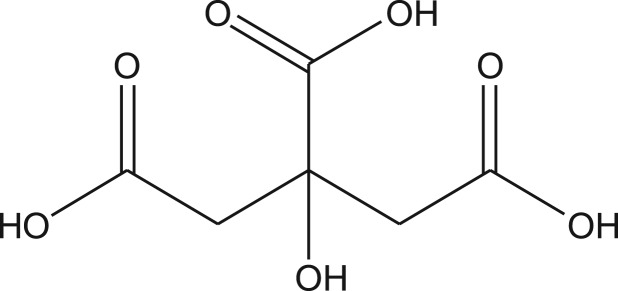
The chemical structure of citric acid.

Citric acid is a common organic acid which has good chelating ability towards metal ions. It has been widely used as a cleaning agent for removing metal ions. The chemical structure of citric acid is shown in figure 1. The hydroxyl group and carboxyl groups in the structure of citric acid are the active groups which account for its good metal-chelating ability. These groups could also be used as linking groups for loading citric acid on an insoluble macromolecule supporter. In recent years, some citric acid-modified plant substrates were synthesized and used to adsorb heavy metal ions. For instance, citric acid-modified *Ceiba pentandra* hulls, soya bean straw and pine sawdust exhibited good abilities to adsorb Cd(II), Cu(II), Ni(II), Pb(II) or Zn(II) ions [11–14]. These results revealed that citric acid is an ideal molecule that could be used as functional group for the synthesis of new adsorbents with considerable adsorption capacities towards metal ions. Besides plant substrates, polystyrene resins are other common support materials which have the advantages of stable mechanical properties, controllable pore structure, good permeability, low cost and easy recovery. However, to date, loading citric acid on resins has not been reported. Compared with traditional weak acid resins, the citric acid-modified resins may have several advantages. The three carboxyl groups in the structure of citric acid would be conducive to enhance metal-chelating ability towards highly charged metal ions. Furthermore, the lone pair electrons in linking atoms (O or N) are also conducive to enhance metal-chelating ability towards metal ions. Thus, loading citric acid on resins is promising to obtain new chelating resins with considerable adsorption capacities towards metal ions.

In this work, new types of chelating resins were synthesized by anchoring citric acid on polystyrene resins. The adsorption behaviours of citric acid-modified resins towards metal ions (Fe^3+^, Al^3+^, Pb^2+^, Cu^2+^, Cd^2+^ and Hg^2+^) were also investigated. The results revealed that PS-CA was a good adsorbent to Fe^3+^, Cu^2+^, Pb^2+^ and Cd^2+^ with *q_m_* values of 143.9 mg g^−1^, 77.4 mg g^−1^, 18.9 mg g^−1^ and 119.9 mg g^−1^, respectively. PS-N-CA was a good adsorbent to Al^3+^ and Hg^2+^ with *q_m_* values of 176.6 mg g^−1^ and 114.9 mg g^−1^, respectively. The desorption experiments indicated that these resins exhibited considerable reusability.

## 2. Experimental

### 2.1. Chemical reagents, adsorbent and samples

Chloromethylated polystyrene resin (PS-Cl) and D380 (PS-NH_2_) were purchased from Chemical Factory of Nankai University (Tianjin, China). Citric acid, trimethyl citrate, KI, NaH, tetrabutylammonium bromide (TBAB), 4-dimethylaminopyridine (DMAP), *N*,*N*-dicyclohexylcarbodiimide (DCC) and 1-hydroxybenzotriazole (HOBt) were purchased from Adamas Reagent Co., Ltd. The reagents and chemicals (analytic grade unless stated otherwise) were purchased from Sinopharm Chemical Reagent Co., Ltd and Tianjin Damao Chemical Reagent Co., Ltd (China).

### 2.2. Synthesis and characterization of citric acid-loaded resins

**Figure 2.**
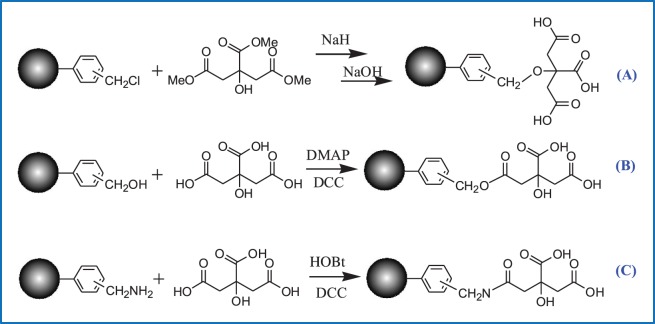
Schemes for the preparation of chelating resins with citric acid (A, PS-CA; B, PS-O-CA; C, PS-N-CA).

Schemes for the preparation of citric acid-modified resins are shown in figure 2. PS-CA, PS-O-CA and PS-N-CA were all synthesized in a three-necked flask equipped with a mechanical stirrer, a thermometer and a reflux condenser. In addition, TBAB and KI were employed as phase transfer catalysts in the reaction. Route A: PS-CA was synthesized according to the following process. Trimethyl citrate (5.6 g) was dissolved in 30 ml dry dimethylformamide (DMF) and then reacted with enough NaH until there were no bubbles [15]. The chloromethylated beads PS-Cl (3.6 g) were allowed to swell in 15 ml dry DMF for 24 h. The treated trimethyl citrate and PS-Cl were both added into a flask. The mixture was stirred (100 r.p.m.) and refluxed for 10 h. The obtained resins were washed with water and then refluxed with NaOH solution. After for 4 h, the citric acid-loaded resins (PS-CA) were filtered and then washed with DMF, water and methanol, then dried under vacuum. Route B: PS-O-CA was synthesized according to the following process. The chloromethylated beads PS-Cl (6 g) were stirred (100 r.p.m.) and refluxed with NaOH solution for 10 h. Then, the hydroxyl beads (PS-OH) were filtered and then washed with water and methanol, then dried under vacuum. The dried PS-OH was then reacted with citric acid using DMAP as the catalyst, and DCC as the dehydrating agent. PS-OH (5.5 g), citric acid (7.5 g), DMAP (0.55 g) and DCC (8.2 g) were added into the flask. The mixture was stirred at room temperature for 8 h. Then, the PS-O-CA was filtered and then washed with DMF, water and methanol, then dried under vacuum. Route C: PS-N-CA was synthesized according to the following process. D380 (PS-NH_2_, 7.0 g) was swelled in 10 ml DMF for 24 h. Then, citric acid (8.5 g), HOBt (5.0 g) and DCC (7.0 g) were added. The mixture was stirred at room temperature for 8 h. Then, the PS-N-CA was filtered and then washed with DMF, water and methanol, then dried under vacuum. In order to quantify the individual metal adsorption ability for different functional groups (–OH, –COOH and amine) towards various metals, PS-COOH was synthesized by the oxidation of PS-Cl using potassium permanganate as the oxidant. Briefly, PS-Cl (6 g) resins were stirred (100 r.p.m.) and refluxed with KMnO_4_ (8.5 g) solution for 8 h. Then, the carboxyl beads (PS-COOH) were filtered and then washed with water and methanol, then dried under vacuum.

### 2.3. Calculation of adsorption capacity

The adsorption capacities of the citric acid-modified resins towards metal ions were calculated according to the following equation:

2.1 $$q_{e}=(C_{0}-C_{e})\times \frac{V_{0}}{W},$$

where *q_e_* is the adsorption capacity (mg g^−1^ dry resin) of resin towards metal ions at adsorption equilibrium. *C_0_* and *C_e_* are the initial and equilibrium concentrations of metal ion solutions (mg ml^−1^). *V*_0_ is the volume of the metal ion solutions. *W* is the weight of dry chelating resins used (g).

### 2.4. The influence of pH on adsorption capacities

The influence of pH on adsorption capacities of resins towards metal ions was determined as follows: 50 ml of metal ion solutions (*C*_0_ = 200 μg ml^−1^) with different pH was shaken with pretreated 0.10 g dry chelating resins in a 100 ml stoppered conical flask in an SHA-B incubator (100 r.p.m.) for 24 h. Subsequently, the concentration of metal ions in the adsorption solution was determined by an atomic absorption spectrophotometer (AAnalyst300).

### 2.5. Adsorption kinetics of citric acid-modified resins towards metal ions

The evaluation of kinetics is of benefit for the prediction of adsorption time and sample concentration. In this study, the adsorption kinetic curves of metal ions on the citric acid-modified resins were obtained as follows: 50 ml of metal ion solutions (*C*_0_ = 200 μg ml^−1^) was shaken with pretreated 0.10 g dry chelating resins in a 100 ml stoppered conical flask in the SHA-B incubator (100 r.p.m.). Subsequently, the concentration of metal ions in the adsorption solution was determined at different times until equilibrium.

### 2.6. Adsorption isotherms of chelating resins towards metal ions

The equilibrium adsorption isotherms of metal ions on the chelating reins were obtained as follows: solutions (50 ml) with different concentrations of metal ions (*C*_0_ = 25, 50, 100, 150 and 200 μg ml^−1^) were contacted with 0.10 g dry chelating resins in conical flasks. The flasks were continually shaken for 2 h (100 r.p.m.). Then, the concentrations of metal ions in the adsorption solutions were determined.

## 3. Results and discussion

### 3.1. Characterization of the modified resins

**Figure 3.**
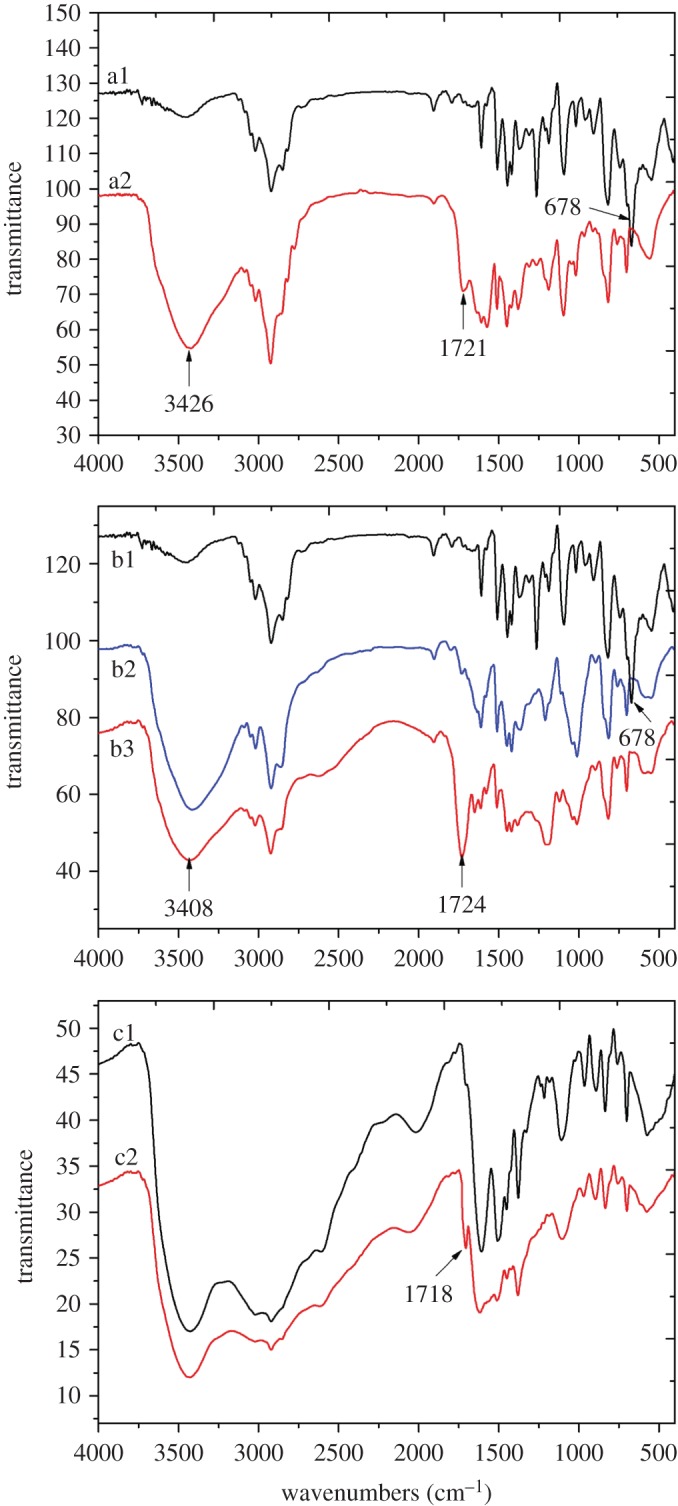
The FTIR spectra of PS-Cl (a1, b1), PS-CA (a2), PS-OH (b2), PS-O-CA (b3), PS-NH_2_ (c1) and PS-N-CA (c2).

Curves a1 and a2 in figure 3 are the Fourier transform infrared (FTIR) spectra of PS-Cl and PS-CA, respectively. In the spectrum of PS-Cl (a1), there was an absorption band in the vicinity of 678 cm^−1^ that was the stretching vibrations of the C–Cl bond [16]. In the spectrum of PS-CA (a2), the carboxyl groups were disclosed by the absorption peaks at 1721 cm^−1^ and 3426 cm^−1^, which were the stretching vibration bands of –C=O and –OH, respectively. This result revealed that citric acid was successfully anchored on resins. In addition, the absorption band in the vicinity of 678 cm^−1^ still existed in curves a1, which indicated that the –Cl groups were not fully substituted by citric acid during the reaction [17,18]. Curves b1, b2 and b3 in figure 3 are the FTIR spectra of PS-Cl, PS-OH and PS-O-CA, respectively. Comparing with the curve of PS-Cl (b1), the new absorption peak of 3408 cm^−1^ in curve b2 was the stretching vibration band of –OH. This result indicated that PS-OH was successfully obtained on the basis of PS-Cl. The absorption peak at 1724 cm^−1^ in curve b3 was the stretching vibration band of *−*C=O revealing that citric acid was successfully anchored on resins through esterification. Curves c1 and c2 in figure 3 are the FTIR spectra of PS-NH_2_ and PS-N-CA, respectively. Comparing with curve c1, the absorption peak at 1718 cm^−1^ was the stretching vibration band of *−*C=O. This result implied that citric acid was successfully anchored on resins through amidation. Based on the gravimetric method, the contents of citric acid in PS-CA, PS-O-CA and PS-N-CA were 0.84 mmol g^−1^, 0.79 mmol g^−1^ and 0.93 mmol g^−1^, respectively.

**Figure 4.**
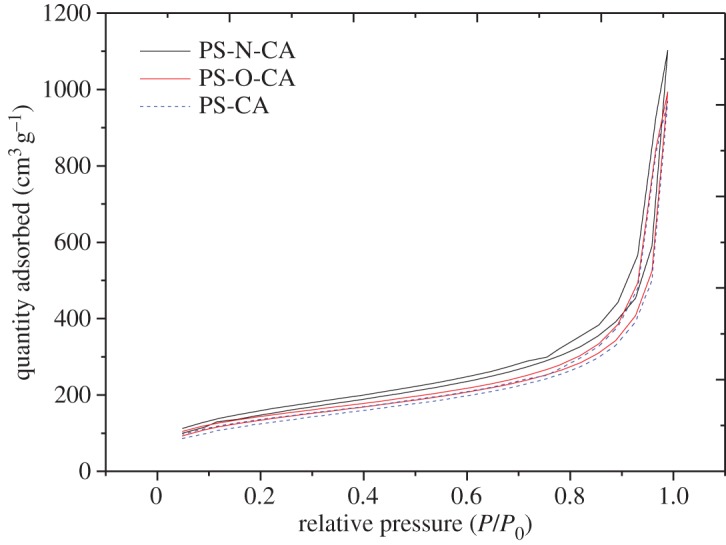
Nitrogen adsorption/desorption isotherms of PS-CA, PS-O-CA and PS-N-CA.

Physical properties of citric acid-modified resins were determined by N_2_ adsorption/desorption isotherms at 77 K using an ASAP 2020 automatic surface area and porosity analyser. The Brunauer–Emmett–Teller (BET) surface area was obtained by the BET method. The pore volume and average pore diameter were obtained by the Barrett–Joyner–Halenda method. Figure 4 shows the adsorption/desorption isotherms of PS-CA, PS-O-CA and PS-N-CA. Physical properties of PS-CA, PS-O-CA and PS-N-CA are tabulated in table 1. The results showed that the physical properties of PS-CA and PS-O-CA were similar in terms of BET surface areas (418.4 m^2^ g^−1^ and 433.9 m^2^ g^−1^, respectively). However, the BET surface area value of PS-N-CA was much higher (524.1 m^2^ g^−1^). This difference is probably due to the fact that the particle sizes of PS-N-CA (0.20–0.35 mm) were much smaller than those of PS-CA and PS-O-CA (0.45–0.60 mm).

**Table 1.** Physical properties of PS-CA, PS-O-CA and PS-N-CA.

resin series	PS-CA	PS-O-CA	PS-N-CA
BET surface area (m^2^ g^−1^)	418.4	433.9	524.1
average pore diameter (nm)	13.9	13.1	11.2
pore volume (cm^3^ g^−1^)	1.54	1.60	1.67
particle size (mm)	0.45–0.60	0.45–0.60	0.20–0.35

### 3.2. The influence of pH on adsorption capacities

**Figure 5.**
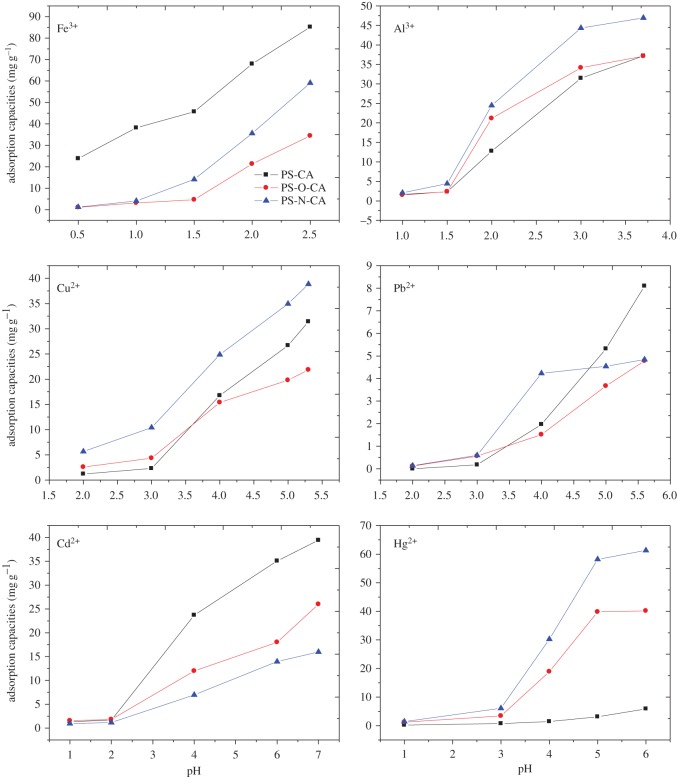
The influence of pH on adsorption capacities of resins towards metal ions.

The influence of pH on adsorption capacities of resins towards metal ions was noted and the results are shown in figure 5. Considering that metal ions would be hydrolysed in high pH, the experiments were carried out at low pH values where metal hydroxide chemical precipitation does not occur. Thus, the pH of Fe^3+^, Al^3+^, Pb^2+^, Cu^2+^, Cd^2+^ and Hg^2+^ was evaluated in ranges of 0.5–2.5, 1.0–3.7, 2.0–5.6, 2.0–5.3, 1.0–7.0 and 1.0–6.0, respectively. From figure 5, it is obvious that the adsorption capacities of resins towards metal ions increased with the increase of pH. This is mainly for the reason that carboxyl groups in citric acid-modified resins existed as carboxylic ions in high pH. Carboxylic ions have good combining abilities towards metal ions. Thus, high pH is conducive to increase the adsorption capacities of metal ions on resins. However, metal ions would be hydrolysed in an excessively high pH solution. After evaluation, the optimal values of pH for Fe^3+^, Al^3+^, Pb^2+^, Cu^2+^, Cd^2+^ and Hg^2+^ were 2.5, 3.7, 5.6, 5.3, 7.0 and 6.0, respectively. These optimal values of pH were adopted in the following adsorption experiments.

In order to quantify the individual metal adsorption ability for different functional groups (−OH, −COOH and amine) towards various metals, the adsorption capacities of metal ions on PS-Cl, PS-OH, PS-NH_2_ and PS-COOH were also investigated at their optimal pH. The results are tabulated in table 2. There were no functional groups and heteroatoms with lone pair electrons in PS-Cl. Thus, PS-Cl exhibited poor ability to adsorb Fe^3+^, Al^3+^, Pb^2+^, Cu^2+^, Cd^2+^ and Hg^2+^ and the adsorption capacities were about 4.16 mg g^−1^, 1.38 mg g^−1^, 1.93 mg g^−1^, 0 g g^−1^, 0 mg g^−1^ and 0.37 mg g^−1^, respectively. In PS-OH and PS-NH_2_, the lone pair electrons in oxygen and in nitrogen have good ability to combine with metal ions. Thus, the adsorption capacities of PS-OH and PS-NH_2_ were higher than those of PS-Cl. In PS-COOH, the carboxyl groups have good ability to combine with metal ions. Thus, the adsorption capacities of resins showed marked improvement after functionalization with –COOH. Comparing the adsorption capacities between PS-Cl, PS-OH, PS-NH_2_, PS-COOH and citric acid-modified resins, it is obvious that the adsorption capacities of metal ions on resins were enhanced measurably after introducing carboxyl groups. In citric acid-modified resins, there are more carboxyl groups and atoms (O or N) with lone pair electrons. Thus, citric acid-modified resins showed the highest adsorption capacities towards metal ions.

**Table 2.** Adsorption capacities of metal ions on PS-Cl, PS-OH, PS-NH_2_ and PS-COOH.

	adsorption capacities (mg g^−1^)					
resins	Fe^3+^	Al^3+^	Pb^2+^	Cu^2+^	Cd^2+^	Hg^2+^
PS-Cl	4.16	1.38	1.93	≈0	≈0	0.37
PS-OH	6.29	4.18	≈0	2.16	5.97	4.42
PS-NH_2_	17.06	3.10	0.24	13.72	7.84	11.97
PS-COOH	37.94	20.71	6.33	18.64	17.89	18.17

### 3.3. Adsorption kinetics of citric acid-modified resins towards metal ions

**Figure 6.**
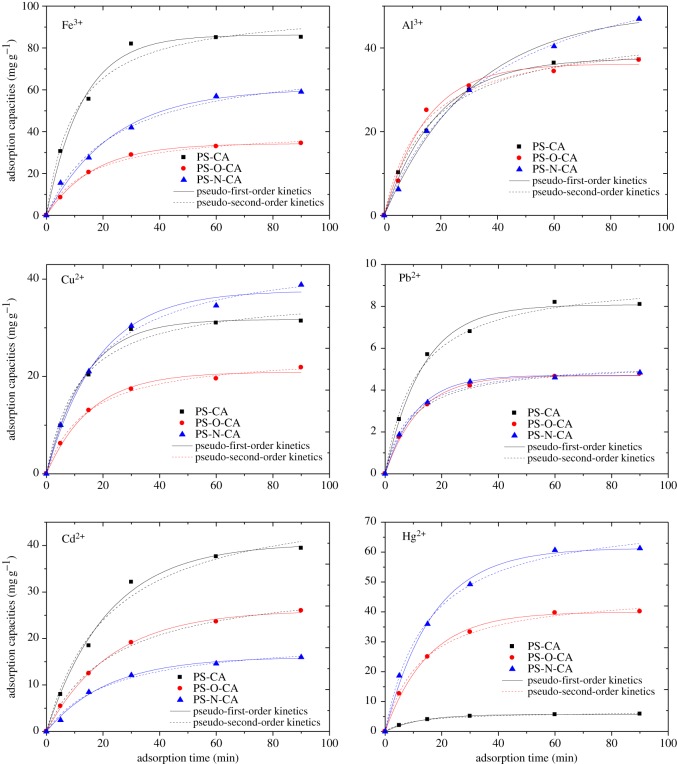
Adsorption kinetics of citric acid-loaded resins towards metal ions.

Adsorption kinetics of PS-CA, PS-O-CA and PS-N-CA towards Fe^3+^, Al^3+^, Pb^2+^, Cu^2+^, Cd^2+^ and Hg^2+^ were all investigated and the results are shown in figure 6. The adsorption capacities of metal ions on PS-CA, PS-O-CA and PS-N-CA were all increased sharply in the first 40 min and then became slow until equilibrium and all of the adsorptions reached equilibrium within 90 min. By comparing the adsorption kinetic curves of Fe^3+^, it can be concluded that PS-CA showed the highest adsorption capacity with a *q_e_* value of about 86.2 mg g^−1^. PS-O-CA showed poor adsorption capacity with *q_e_* value of about 34 mg g^−1^. The adsorption capacities of Fe^3+^ on resins followed the order of PS-CA > PS-N-CA > PS-O-CA. This result indicated that the three carboxyl groups in PS-CA played an important role in the adsorption of Fe^3+^. The trends of adsorption kinetic curves of Al^3+^ indicated that the adsorption capacities of Al^3+^ on resins followed the order of PS-N-CA > PS-CA ≈ PS-O-CA. The nitrogen atom in PS-N-CA plays an important role in combining with Al^3+^. For the adsorption behaviours of Cu^2+^, PS-N-CA showed the highest adsorption capacity with a *q_e_* value of about 38 mg g^−1^. But, PS-O-CA showed the lowest adsorption capacity with a *q_e_* value of about 21 mg g^−1^. The nitrogen atom also plays an important role in combining with Cu^2+^. PS-CA, PS-O-CA and PS-N-CA all showed poor adsorption capacities towards Pb^2+^. The values of *q_e_* for them were 8.1 mg g^−1^, 4.6 mg g^−1^ and 4.7 mg g^−1^, respectively. The trends of adsorption kinetics curves of Cd^2+^ followed the order of PS-CA > PS-O-CA > PS-N-CA. The values of *q_e_* for them were 40.1 mg g^−1^, 26.0 mg g^−1^ and 16.0 mg g^−1^, respectively. The adsorption capacities of resins towards Hg^2+^ exhibited big differences with *q_e_* for PS-O-CA and PS-N-CA being 40.0 mg g^−1^ and 61.4 mg g^−1^, respectively, while that for PS-CA was 5.7 mg g^−1^. This result indicated that lone pair electrons in oxygen and in nitrogen play an important role in the adsorption of Hg^2+^. Overall, most citric acid-modified resins were good for the adsorption of Fe^3+^, Al^3+^, Cu^2+^, Cd^2+^ and Hg^2+^ but poor for the adsorption of Pb^2+^.

In order to better illustrate the adsorption mechanisms of metal ions on modified resins, pseudo-first-order and pseudo-second-order kinetic models were employed to fit the above experimental data. The model that performed best was selected on the basis of the linear regression correlation coefficient values (*R*^2^). These two kinetic equations are presented as follows. Equation of pseudo-first-order kinetic model [19]:

3.1 $$\ln(q_{e}-q_{t})=\ln q_{e}-k_{1}t.$$

Equation of pseudo-second-order kinetic model [20]:

3.2 $$\frac{t}{q_{t}}=\frac{1}{k_{2}{q_{e}}^{2}}+\frac{1}{q_{e}}t,$$

where *q_e_* and *q_t_* are the adsorption capacity of metal ions on the citric acid-modified resins at equilibrium and at any time *t* (mg g^−1^ dry resin), respectively. The parameters *k*_1_ (min^−1^) and *k_2_* (g mg^−1^ min^−1^) are the rate constants of the pseudo-first-order and pseudo-second-order models for the adsorption process, respectively.

The kinetic data in figure 6 were fitted by the above two models. The calculated results of the pseudo-first-order and pseudo-second-order rate equations are shown in table 3. The parameter *R*^2^ was adopted to evaluate the models. The results showed that the values of *R*^2^ for pseudo-first-order and pseudo-second-order rate equations were mostly above 0.98, which indicated that these two models could be used to fit the adsorption processes. The values of *R*^2^ for the pseudo-first-order rate equation were mostly higher than 0.99, which showed the good quality of linearization. Thus, the pseudo-first-order kinetic equation was more appropriate for the adsorption. Based on the *q_e_* calculated by pseudo-first-order rate equation, it can be concluded that PS-CA was a good adsorbent to absorb Fe^3+^ and Cd^2+^, PS-N-CA was a good adsorbent to adsorb Al^3+^, Cu^2+^ and Hg^2+^. PS-CA, PS-O-CA and PS-N-CA all showed poor abilities to adsorb Pb^2+^ with *q_e_* below 10 mg g^−1^.

**Table 3.** Values of kinetic parameters for adsorption of metal ions.

		pseudo-first-order equation			pseudo-second-order equation		
metal ions	resins	*k_1_* (min^−1^)	*q_e_* (mg g^−1^)	*R^2^*	*k_2_* (g mg^−1^ min^−1^)	*q_e_* (mg g^−1^)	*R^2^*
Fe^3+^	PS-CA	0.079	86.20	0.9919	0.00097	99.45	0.9807
	PS-O-CA	0.060	34.34	0.9993	0.00156	41.44	0.9925
	PS-N-CA	0.043	60.61	0.9906	0.00055	76.36	0.9925
Al^3+^	PS-CA	0.054	37.57	0.9973	0.00126	45.70	0.9953
	PS-O-CA	0.068	36.22	0.9872	0.00169	43.34	0.9782
	PS-N-CA	0.032	48.80	0.9959	0.00042	65.76	0.9969
Cu^2+^	PS-CA	0.074	31.74	0.9955	0.0023	37.07	0.9813
	PS-O-CA	0.064	20.91	0.9920	0.0029	24.94	0.9971
	PS-N-CA	0.055	37.63	0.9934	0.0013	45.83	0.9966
Pb^2+^	PS-CA	0.076	8.08	0.9933	0.0095	9.41	0.9921
	PS-O-CA	0.084	4.68	0.9969	0.0194	5.38	0.9977
	PS-N-CA	0.091	4.71	0.9961	0.0216	5.36	0.9948
Cd^2+^	PS-CA	0.046	40.45	0.9939	0.00085	51.24	0.9847
	PS-O-CA	0.044	26.07	0.9985	0.00127	33.05	0.9984
	PS-N-CA	0.046	16.01	0.9933	0.00216	20.32	0.9884
Hg^2+^	PS-CA	0.082	5.74	0.9979	0.01496	6.63	0.9967
	PS-O-CA	0.066	39.98	0.9963	0.00162	47.15	0.9977
	PS-N-CA	0.059	61.39	0.9947	0.00092	73.24	0.9970

### 3.4. Adsorption isotherms of citric acid-modified resins towards metal ions

**Figure 7.**
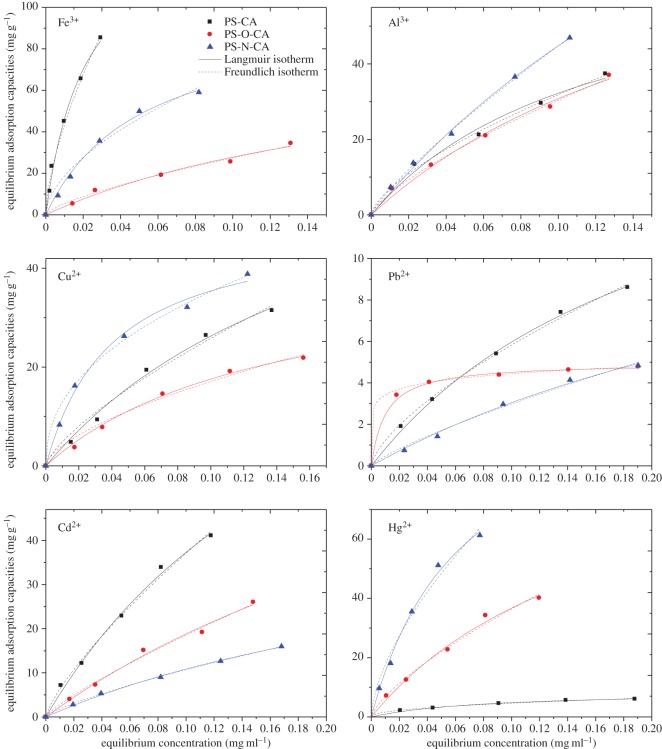
Adsorption isotherms of citric acid-loaded resins towards metal ions.

The equilibrium adsorption isotherms of metal ions on citric acid-modified resins were obtained at initial concentrations of 25, 50, 100, 150 and 200 μg ml^−1^. The obtained results are shown in figure 7. It is obvious that the adsorption capacities of resins increased with increasing concentration of metal ions. By comparing the adsorption isotherm curves of Fe^3+^, the adsorption capacities of Fe^3+^ on resins followed the order of PS-CA > PS-N-CA > PS-O-CA. The trends of adsorption curves of Al^3+^ indicated that the adsorption capacities of Al^3+^ on resins followed the order of PS-N-CA > PS-CA ≈ PS-O-CA. The nitrogen atom plays an important role in combining with Al^3+^. For the adsorption behaviours of Cu^2+^, PS-N-CA showed the highest adsorption capacity, while PS-O-CA showed the lowest adsorption capacity. The nitrogen atom also plays an important role in combining with Cu^2+^. PS-CA, PS-O-CA and PS-N-CA all showed poor adsorption capacities towards Pb^2+^. The trends of adsorption kinetics curves of Cd^2+^ followed the order of PS-CA > PS-O-CA > PS-N-CA. The adsorption capacities of resins towards Hg^2+^ have exhibited big difference. PS-N-CA showed a good adsorption behaviour towards Hg^2+^.

In order to understand the adsorption mechanism of metal ions on citric acid-modified resins, Langmuir and Freundlich models have been employed to explain the process of adsorption equilibrium. The results are shown in table 4. The Langmuir isotherm equation and Freundlich isotherm equation are represented as equations (3.3) and (3.4), respectively [21,22]:

3.3 $$\frac{C_{e}}{q_{e}}=\frac{K_{L}}{q_{m}}+\frac{C_{e}}{q_{m}}$$

and

3.4 $$\mathrm{lg}q_{e}=\left(\frac{1}{n}\right)\mathrm{lg}C_{e}+\mathrm{lg}K_{F},$$

where *q_e_* and *q_m_* are the equilibrium and maximum adsorption capacity (mg g^−1^ dry resin), respectively. *C_e_* is the equilibrium concentration of metal ion solution (mg ml^−1^). *K_L_* is the parameter related to the adsorption energy (ml mg^−1^). *K_F_* reflects the adsorption capacity of resins ((mg g^−1^)(ml mg^−1^)^1/*n*^). The parameter *n* represents the adsorption affinity of the adsorbent for resins.

**Table 4.** Langmuir and Freundlich isotherm parameters for the adsorption processes of metal ions on resins.

		Langmuir			Freundlich		
metal ions	resins	*q_m_* (mg g^−1^)	*K_L_* (ml mg^−1^)	*R^2^*	*K_F_* ((mg g^−1^) (ml mg^−1^)^1/*n*^)	*1/n*	*R^2^*
Fe^3+^	PS-CA	143.97	0.021	0.9859	811.02	0.63	0.9835
	PS-O-CA	83.14	0.199	0.9843	140.30	0.71	0.9817
	PS-N-CA	97.45	0.051	0.9901	252.46	0.57	0.9795
Al^3+^	PS-CA	71.01	0.120	0.9833	146.73	0.66	0.9890
	PS-O-CA	92.84	0.201	0.9875	163.75	0.73	0.9873
	PS-N-CA	176.59	0.294	0.9848	296.44	0.82	0.9862
Cu^2+^	PS-CA	77.41	0.195	0.9904	137.44	0.73	0.9818
	PS-O-CA	41.67	0.137	0.9911	71.81	0.62	0.9823
	PS-N-CA	49.64	0.040	0.9869	96.55	0.44	0.9894
Pb^2+^	PS-CA	18.93	0.217	0.9913	27.70	0.68	0.9863
	PS-O-CA	4.97	0.010	0.9925	5.76	0.11	0.9917
	PS-N-CA	15.82	0.419	0.9907	18.89	0.80	0.9781
Cd^2+^	PS-CA	119.96	0.222	0.9911	218.63	0.77	0.9879
	PS-O-CA	95.40	0.407	0.9857	123.21	0.82	0.9783
	PS-N-CA	48.55	0.347	0.9936	62.99	0.77	0.9929
Hg^2+^	PS-CA	8.78	0.082	0.9919	13.39	0.46	0.9868
	PS-O-CA	99.99	0.172	0.9877	192.57	0.72	0.9806
	PS-N-CA	114.98	0.065	0.9885	315.53	0.62	0.9764

The equilibrium data in figure 7 were fitted by the above two isotherm models. The corresponding parameters of Langmuir model and Freundlich model were calculated and are tabulated in table 4. The values of *R^2^* were employed to evaluate the models. The results showed that the values of *R^2^* for the Langmuir model were all above 0.98, indicating that the Langmuir model was more appropriate for the adsorption. Based on the *q_m_* calculated by Langmuir model equation, the maximum adsorption capacities of metal ions on PS-CA, PS-O-CA and PS-N-CA were obtained. PS-CA showed good adsorption capacities towards Fe^3+^, Cu^2+^, Pb^2+^ and Cd^2+^ with *q_m_* values of 143.9 mg g^−1^, 77.4 mg g^−1^, 18.9 mg g^−1^ and 119.9 mg g^−1^, respectively. PS-N-CA showed good adsorption capacities towards Al^3+^ and Hg^2+^ with *q_m_* values of 176.6 mg g^−1^ and 114.9 mg g^−1^, respectively. PS-CA, PS-O-CA and PS-N-CA have different adsorption effects on metal ions. These results indicated that the lone pair electrons in linking atoms (O or N) play an important role in the adsorption. Different materials may have different active sites to combine with metal ions. Further investigations are being conducted to give an explanation of this phenomenon.

### 3.5. Comparison of adsorption capacities between different citric acid-modified adsorbents

A comparison of the results of citric acid-modified resins obtained in this work with those of some other adsorbents reported in the literature is given in table 5 [11–13]. For copper ion, the *q*_max_ values of CA-SD, CA-BWSS, CA-WWSS and CA-PS were 15.06 mg g^−1^, 48.90 mg g^−1^, 48.26 mg g^−1^ and 16.19 mg g^−1^, respectively. In our work, citric acid-modified resins exhibited good abilities to adsorb Cu^2+^ with *q*_max_ values for PS-CA, PS-O-CA and PS-N-CA of 77.41 mg g^−1^, 41.67 mg g^−1^ and 49.64 mg g^−1^, respectively. The *q*_max_ values of Cd^2+^ on citric acid-modified resins were much higher than those on CA-SD, CA-BWSS, CA-WWSS and CA-PS. While the *q*_max_ values of Pb^2+^ on citric acid-modified resins were much lower than those on citric acid-modified plant substrates. The above results indicated that the insoluble carrier in citric acid-modified adsorbents also has great influence on adsorption. Polystyrene resins have large surface area and good permeability. Thus, the adsorption capacities of Cu^2+^ and Cd^2+^ on citric acid-modified resins were much higher. However, plant substrates also have some advantages. For instance, plant fibre is a highly polar substance which contains very high levels of –OH groups. This property benefits the adsorption behaviours of adsorbents towards metal ions. It is probably for this reason that the adsorption capacities of Pb^2+^ on citric acid-modified plant substrates were much higher.

**Table 5.** Citric acid-modified adsorbents used for the adsorption of metal ions. CA-SD, CA modified sawdust; CA-BWSS, CA modified base washed soya bean straw; CA-WWSS, CA modified water washed soya bean straw; CA-PS, CA-modified pine sawdust.

	*q*_max_ calculated from Langmuir model (mg g^−1^)						
adsorbents	Fe^3+^	Al^3+^	Pb^2+^	Cu^2+^	Cd^2+^	Hg^2+^	references
CA-SD	—	—	48.48	15.06	12.81	—	[11]
CA-BWSS	—	—	—	48.90	—	—	[12]
CA-WWSS	—	—	—	48.26	—	—	[12]
CA-PS	—	—	>51.8	16.19	>22.45	—	[13]
PS-CA	143.97	71.01	18.93	77.41	119.96	8.78	our study
PS-O-CA	83.14	92.84	4.97	41.67	95.40	99.99	our study
PS-N-CA	97.45	176.59	15.82	49.64	48.55	114.98	our study

### 3.6. Desorption of metal ions adsorbed on citric acid-modified resins

Desorption tests of removing metal ions on citric acid-modified resins were also conducted. According to the results in figure 5, it can be concluded that reduction of pH would make carboxyl group non-ionized and decrease the combining capacities between resins and metal ions. Thus, metal ions adsorbed on resins might be desorbed by shaking with acidic solutions. In our experiments, metal ions adsorbed on resins were desorbed by shaking with 20 ml acidic solution for 24 h. The results showed that most of the metal ions could be desorbed efficiently with desorption ratios above 98% in 0.5% HCl solution. However, the desorption ratio of Fe^3+^ on PS-CA was 74.6%. With the enhancement of acidity, Fe^3+^ on PS-CA was desorbed efficiently (greater than 98%) in 3.0% HCl concentration. Overall, metal ions adsorbed on citric acid-modified resins could be desorbed efficiently by shaking with HCl solution. This result also suggested that citric acid-modified resins could be regenerated using 0.5% or 3.0% HCl solution.

### 3.7. Recycling ability of the citric acid-modified resins

**Figure 8.**
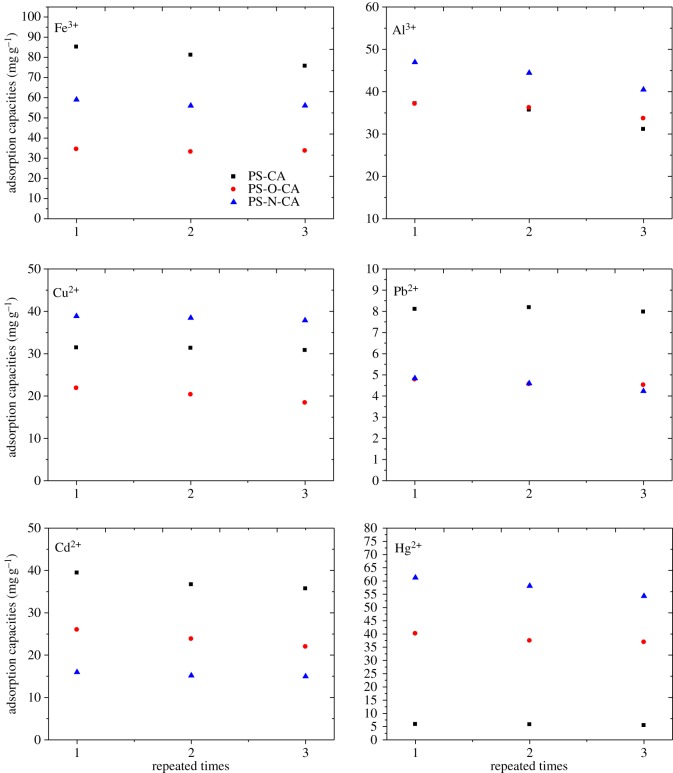
Recycling ability of the citric acid-loaded resins towards metal ions.

PS-CA, PS-O-CA and PS-N-CA were repeatedly used three times for the continuous adsorption and desorption of metal ions. The adsorption capacities of resins for every time are shown in figure 8. After being reused for three times, all of the resins showed good adsorption abilities. For instance, the adsorption capacities of PS-CA towards Fe^3+^ at three times were 85.15 mg g^−1^, 81.17 mg g^−1^ and 75.67 mg g^−1^, respectively. The results revealed that PS-CA, PS-O-CA and PS-N-CA exhibited considerable recyclability.

## 4. Conclusion

In summary, citric acid-modified resins PS-CA, PS-O-CA, PS-N-CA were synthesized by anchoring citric acid on PS-Cl, PS-OH and PS-NH_2_, respectively. These citric acid-modified resins exhibited good abilities to adsorb Fe^3+^, Al^3+^, Cu^2+^, Cd^2+^ and Hg^2+^, while poor abilities to adsorb Pb^2+^. The adsorption capacities of metal ions on resins were significantly influenced by pH. The ideal values of pH for Fe^3+^, Al^3+^, Pb^2+^, Cu^2+^, Cd^2+^ and Hg^2+^ were 2.5, 3.7, 5.6, 5.3, 7.0 and 6.0, respectively. The adsorption kinetics and adsorption isotherm experiments indicated that the pseudo-first-order rate equation was more appropriate for characterizing the kinetic data and the Langmuir model was more suitable for fitting the equilibrium data. PS-CA, PS-O-CA and PS-N-CA have different adsorption effect for metal ions. Further investigations are being conducted to give an explanation of this phenomenon. In the desorption experiments, Fe^3+^, Al^3+^, Pb^2+^, Cu^2+^, Cd^2+^ and Hg^2+^ on resins were efficiently desorbed by shaking with HCl solution with desorption ratios above 98%. After desorption, these resins still exhibited considerable adsorption abilities.

## Data accessibility

All data are provided in the main text.
